# Investigating immune and non‐immune cellular profiles in recurrent respiratory papillomatosis by multi‐omics

**DOI:** 10.1002/ctm2.1570

**Published:** 2024-03-01

**Authors:** Zijie Niu, Yang Xiao, Yiran Li, Sihan Zhou, Meiyu Liu, Fangyuan Li, Yaran Zhang, Jun Wang, Xunyao Wu

**Affiliations:** ^1^ Department of Otorhinolaryngology‐Head and Neck Surgery Beijing Tongren Hospital, Capital Medical University Beijing China; ^2^ Key Laboratory of Otolaryngology‐Head and Neck Surgery Ministry of Education Beijing China; ^3^ Clinical and Science Investigation Institute Peking Union Medical College Hospital, Chinese Academy of Medical Sciences and Peking Union Medical College Beijing China; ^4^ State Key Laboratory of Complex Severe and Rare Diseases Peking Union Medical College Hospital, Chinese Academy of Medical Science and Peking Union Medical College Beijing China

Dear Editor,

1

Recurrent respiratory papillomatosis (RRP) is an unusual benign tumour featuring the perennial proliferation of exophytic papilloma in the upper aerodigestive tract.^1^, ^2^ No curative therapy, no effective prevention, and a high economic burden for families highlight the importance and urgency of exploring RRP pathogenesis. In this study, we performed multi‐omics analysis for cell heterogeneity of tumour microenvironment (TME) and would provide a valuable source for developing new targets (Figure 1A).

**FIGURE 1:**
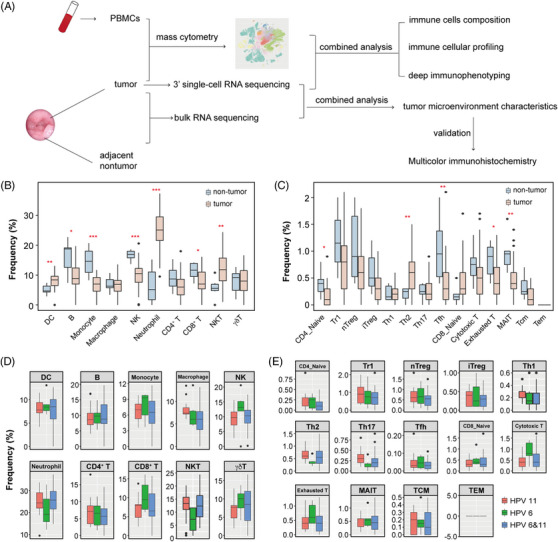
Analysis of immune cell abundance in bulk RNA‐seq data. (A) Workflow of the overall study. PBMCs: peripheral blood mononuclear cells. (B) Comparisons of immune cell frequency between tumours and non‐tumour tissues. DC: Dendritic cell; B: B cell; NK: natural killer cell; NKT: natural killer T cell. (C) Comparisons of T cell subsets frequency between tumours and non‐tumour tissues. nTreg: natural regulatory T cell; iTreg: induced regulatory T cell; Th: T helper; Tfh: follicular helper T; MAIT: mucosal‐associated invariant T; Tcm: central memory T; Tem: effector memory T. (D) Comparison of immune cell frequency among HPV 6 (*n* = 6), HPV 11 (*n* = 13), and HPV 6&11 (*n* = 30) subgroups in tumour tissues of recurrent respiratory papillomatosis (RRP) patients. (E) Comparison of T cell subsets frequency among different subgroups in tumour tissues of RRP patients.The two‐sided Wilcoxon test or Kruskal‐Wallis test was used for each comparison. Endpoints depict the minimum and maximum values. The center lines and whisker denote median values and 1.5 × In the interquartile range, respectively. *: *p* < .05, **: *p* < .01, ***: *p* < .001.

We performed bulk RNA‐seq from 49 RRP tumour tissues and six adjacent normal tissues. The principal component analysis plot distinguished between the tumour and control tissue. We identified 3914 differentially expressed genes (DEGs) and then employed the Kyoto Encyclopedia of Genes and Genomes enrichment analysis (Figure S1). We used ImmunCellAI to estimate and compare the immune cell abundance. We found lower percentages of B cell, monocyte, natural killer cell (NK), CD8^+^ T and higher percentages of dendritic cell (DC), neutrophil, and natural killer T cells (NKT) compared with non‐tumour tissues (Figure 1B). In addition, analysis of T cell subsets showed lower percentages of naïve CD4^+^ T (CD4_Naive), follicular helper T (Tfh), exhausted T (Exhausted T), mucosal‐associated invariant T (MAIT) and a higher percentage of Th2 in tumours (Figure 1C). The percentages of immune cell subsets were not associated with Human Papillomavirus (HPV) phenotyping (Figure 1D,E).

Next, we employed 3′ scRNA‐seq of freshly dissociated tumour samples from six RRP patients. We identified ten major cell types harbored DEGs representing distinct cell type (Figure S2). Examination of feature markers in re‐clustered immune cells revealed eight major cell populations, in which, T cell, macrophage, neutrophil and DC comprised the majority of tumour‐infiltrating lymphocytes (TILs) (Figures S3A,B and S4A). We subset all T cells and divided them into CD8^+^ T/NK and CD4^+^ T cell subsets (Figure S3C,D). We observed that NK and exhausted CD8^+^ T are the major subsets of the CD8 T/NK and Th17, Th2, and Treg are the major components of CD4^+^ T cells (Figure S4B–G). Among re‐clustered myeloid cells, we identified five macrophage subsets, four DC subsets, pDC, neutrophil, mast cell and SERPIB3+ cells (Figure S5A,B). Myeloid cells predominantly comprise macrophage and DC (Figure S5C). For macrophage, Mk_1‐*MMP12*, and Mk_2, expressing *CCL18*, *CD163* and *PCNA*, are enriched in tumours (Figure S5D,E). For DC clusters, DC_1 (*MMP12* and *IL10*) and DC_2 (*PCNA*, *CCL18* and *CCL13*) are dominantly rich in TILs of RRP (Figure S5F,G).

To deeply characterize the immunophenotype of TILs, we performed mass cytometry using a 36‐marker panel for a subset of 24 RRP tumours and 11 matched peripheral blood mononuclear cells (PBMCs). CD45^+^ TILs were visualized, and seven significant populations were characterized (Figure 2A). The most frequent immune cell subtypes observed were, in order, T cell, DC, NK, granulocyte, B cell, macrophage and monocyte (Figure 2B). The growth and production of interferon‐γ (IFN‐γ) of CD4^+^ and CD8^+^ T cells were enhanced in TILs than in PBMCs (Figure 2C). A decreased frequency of granzyme B‐producing NK and CD4^+^T cells was observed. The percentage of PD‐1^+^NK, CD8^+^PD‐1^+^T, CD4^+^ PD‐1^+^T, as well as CD4^+^ CTLA‐4^+^T in TILs was significantly higher than PBMCs (Figure 3D). Higher PD‐1^high^ population had a dramatically higher risk of recrudesce and was related to terrible disease‐free survival in head and neck tumour patients.^3^, ^4^ These findings indicated that the exhausted status of T and NK cells might represent another essential mechanism for immune evasion. Phenotype analysis of DCs showed increased human leukocyte antigen (HLA)‐DR^+^ and IDO1^+^ but decreased percentage of CD40^+^ DC in TILs compared with PBMCs (Figure 2E). Since high‐risk HPV could trigger a regional Th2 inflammation at an incipient period and promote the gradual progression of cervical carcinoma,^5^, ^6^ we inferred that the preferential accumulation of Th2, Treg, and immunosuppressive DCs in TILs might be an essential mechanism for tumour progression in RRP patients.

**FIGURE 2:**
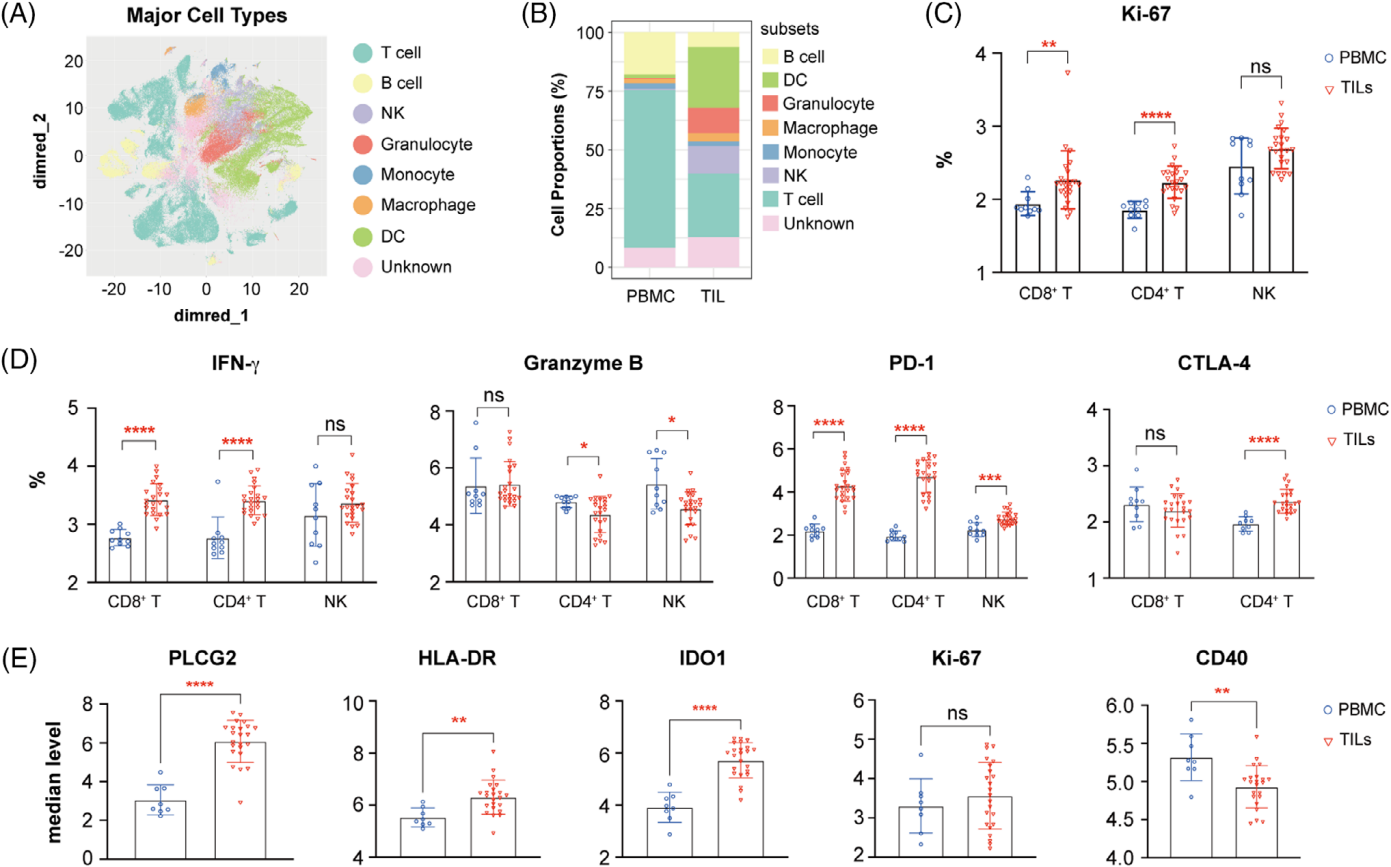
Phenotype analysis of tumour‐infiltrating lymphocytes of recurrent respiratory papillomatosis (RRP) by mass cytometry. (A)The identification of major CD45^+^ cell types from tumours and peripheral blood mononuclear cells (PBMCs) was visualized using t‐SNE. (B) Average abundances of major CD45^+^ cell types from tumours and PBMCs. (C, D) Comparisons of different CD8^+^ T, CD4^+^ T or NK subpopulations between tumours and PBMCs of RRP patients. (E) Comparisons of different dendritic cell (DC) subpopulations between tumours and PBMCs of RRP patients. The two‐sided Wilcoxon test was used for each comparison. Each dot represents an independent individual. ns: not significant, *: *p* < .05, **: *p* < .01, ***: *p* < .001, ****: *p* < .001.

**FIGURE 3:**
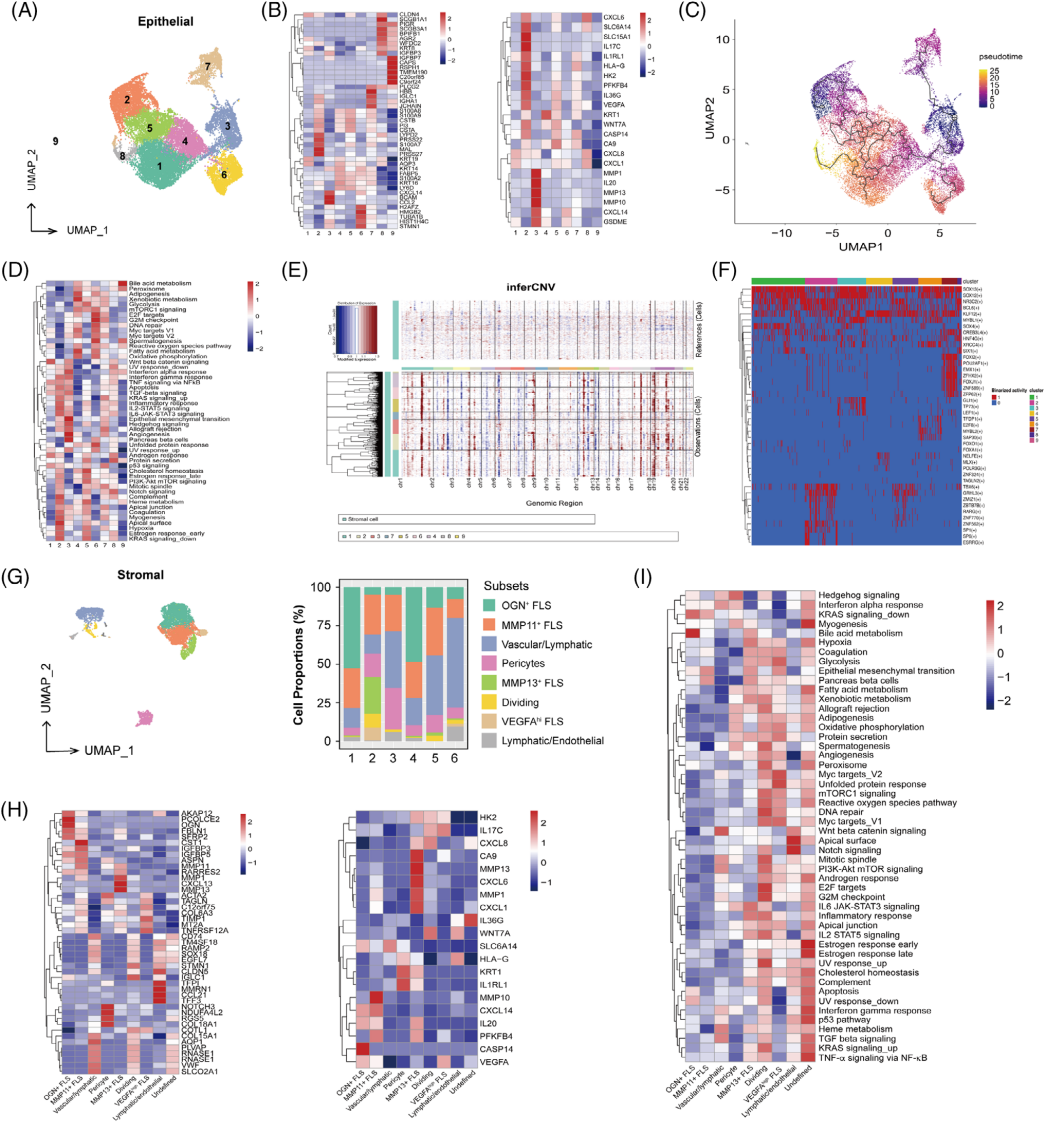
Expression profile and development of non‐immune cells in recurrent respiratory papillomatosis (RRP) patients. (A) UMAP dimensionality reduction of 21 082 epithelial cells was visualized. (B) Heatmap showing the top five gene expressions, interest, and upregulated gene expressions from bulk RNA‐sequencing (RNA‐seq) in each epithelial subcluster. (C) Pseudotime trajectory analysis of all epithelial cells inferred by Monocle 3, in which both cluster 2 and cluster 3 were selected as the start cells. (D) Heatmap showing the representative pathway terms of Hallmark enriched in each epithelial subcluster by gene set variation analysis (GSVA) analysis. (E) The hierarchical heatmap shows the inferred large‐scale copy number variations (CNVs) of epithelial subclusters from all samples. The data from stromal cells were used as references. (F) Heatmap of regulon activity analyzed by SCENIC. The “regulon” refers to the regulatory network of transcriptive factors (TFs) and their target genes. “On” indicates active regulons; “Off” indicates inactive regulons. (G) UMAP dimensionality reduction of 6491 stromal cells was visualized. Bar plot showing the stromal cell abundance within tumours across all samples. (H) Heatmap showing the top five gene expressions, interest, and upregulated gene expressions from bulk RNA‐seq in each stromal subcluster. (I) Heatmap showing the representative pathway terms of Hallmark enriched in each stromal subcluster by GSVA analysis.

To further investigate the carcinogenesis of RRP, we subset non‐immune cells from scRNA‐seq and performed the integrated analysis with data from bulk RNA‐seq (Table S1). Re‐clustering epithelial subset yields nine subsets (Figure 3A). Cluster 2, specifically expressing *LYPD2*, *MAL* and *S100A7*, showed a high level of *IL17C*, *HK2*, *PFKFB4*, *IL36G* and *VEGFA* expressions (Figure 3B). Cluster 3, which featured *CXCL14*, *BCAM* and *CCL2* expressions, showed a high level of *MMP13* and *MMP10* expressions (Figure 3B). Pseudotime trajectory analysis using Monocle3 revealed that compared with cluster 2, cluster 3 is more likely to develop into dividing subsets (Cluster 6_*HMGB2*) (Figure 3B,C). Competitive gene set variation analysis (GSVA) was completed to unveil the biological positions of each cellular cluster in RRP tumourigenicity and advancement. Notably, we observed that “Hypoxia” as well as “IL6‐JAK‐STAT3 signalling” were enrichment in cluster 2. “IFN‐αresponse,” “IFN‐γresponse” and “TGF‐βsignalling” were enrichment in cluster 3, along with “Angiogenesis” and “Epithelial‐mesenchymal transition” (Figure 3D). Large‐scale chromosome copy number variation compared to reference data of stromal cells indicated malignant status across all the identified epithelial subsets (Figure 3E). In addition, we explored the regulons activity by single‐cell regulatory network inference and clustering (SCENIC). *NR3C2*, *BCL6*, *TBX6* and *GRHL3* have been identified as candidate transcriptive factors (TFs) in cluster 2. *SOX13* and *SOX12* were identified as candidate TFs in cluster 3 (Figure 3F).

Combined analysis of the top five DEGs of each stomal cluster in scRNA‐seq data and interested upregulated genes from bulk RNA‐seq data showed that *MMP10* expressed on *MMP11* ^+^ FLS and *MMP13* expressed on *CXCL13* ^+^ *CXCL6* ^+^ *MMP13* ^+^ FLS (Figure 3G,H). However, the enriched inflammatory pathways such as “IL6‐JAK‐STAT3 signalling,” “Hypoxia,” “metabolism‐related pathways” as well as “inflammatory response” were observed in *CXCL13* ^+^ *CXCL6* ^+^ *MMP13* ^+^ FLS but not *MMP11* ^+^ FLS by GSVA analysis (Figure 3I). MMP13 could promote tumour angiogenesis by inducing the secretion of vascular endothelial growth factor A from fibroblast and endothelial cells.^7^, ^8^ MMP10 could also take part in carcinoma angiogenesis and proliferation.^9^ Finally, we validated the presence of CXCL14^+^CCL2^+^MMP13^+^MMP10^+^ in tumours of four RRP patients and CXCL13^+^CXCL6^+^MMP13^+^MMP10^+^ cells in three RRP patients by multicolour immunohistochemistry staining (Figure 4).

**FIGURE 4:**
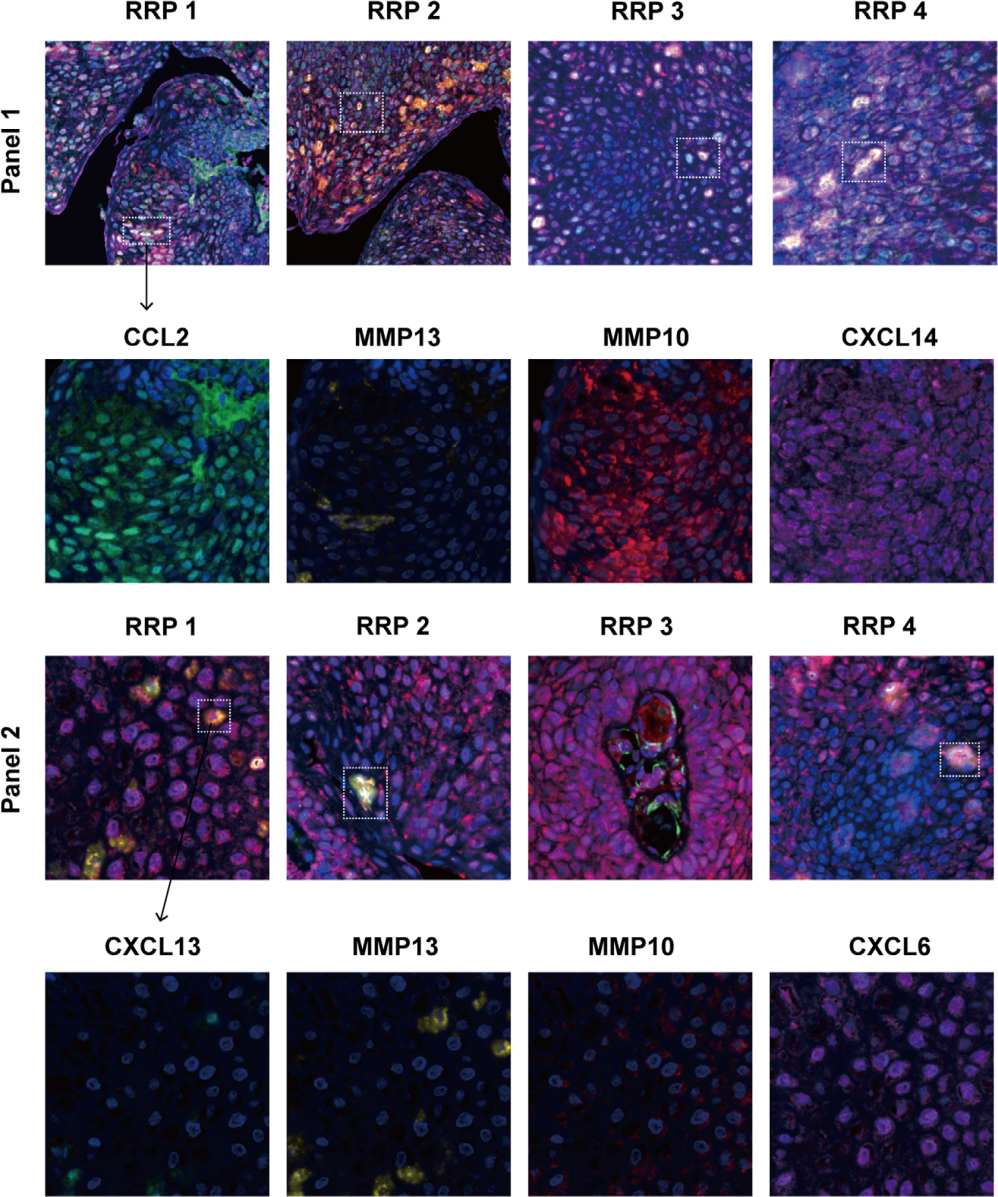
Multicolor immunohistochemistry staining to determine the pathogenic populations in tumours of recurrent respiratory papillomatosis (RRP) patients. Representative images of multicolour immunohistochemistry staining of CCL2^+^CXCL14^+^MMP13^+^MMP10^+^ (Panel 1) and CXCL13^+^CXCL6^+^MMP13^+^MMP10^+^ (Panel 2) on paraffin‐embedded tissue of four RRP tumours. DAPI (blue) was used to visualize cell nuclei. Panel 1: CCL2 (green), MMP13 (yellow), MMP10 (red), CXCL14 (magenta); Panel 2: CXCL13 (green), MMP13 (yellow), MMP10 (red) and CXCL6 (magenta).

Collectively, our work first systematically deciphered the intra‐tumoural immune and non‐immune cell characteristics by multi‐omics. We characterized the immune landscape and identified an MMP13‐ and MMP10‐secreting *CXCL14* ^+^ *CCL2* ^+^ pathogenic epithelial subset within RRP tumours.

## CONFLICT OF INTEREST STATEMENT

The authors declare no conflict of interest.

## ETHICS STATEMENT

The study was approved by the Ethics Committee of Beijing Tongren Hospital, Beijing, China (No. TRECKY2021‐023).

## Supporting information

Supporting Information

Supporting Information

Supporting Information

## Data Availability

The accession number for the sequencing raw data has been deposited in the Genome Sequence Archive for Human (GSA‐Human) under accession number HRA003540. To comply with the “Guidance of the Ministry of Science and Technology (MOST) for the Review and Approval of Human Genetic Resources”, we are required to deposit the genomic data of Chinese people under controlled access at the GSA in Beijing Institute of Genomics Data Center. To gain access to the raw data under accession number HRA003540, please submit requests to the GSA‐Human online page for this study. (https://ngdc.cncb.ac.cn).
