# Supplementary material for: Investigating immune and non‐immune cellular profiles in recurrent respiratory papillomatosis by multi‐omics
Source: Clin Transl Med. 2024 Mar 1;14(3):e1570. doi: 10.1002/ctm2.1570 (PMC10905527; doi:10.1002/ctm2.1570)
Supplement: Supplementary file 1 — Supporting Information [file CTM2-14-e1570-s003.docx]

**Methods**

**Study population**

Tissues were obtained from patients pathologically diagnosed with RRP and underwent surgical interventions at Beijing Tongren Hospital from January 2021 to August 2022. The severity of the disease was assessed using the Derkay score, and HPV genotyping was performed as previously described^[1, 2]^. The study was approved by the Ethics Committee of Beijing Tongren Hospital, Beijing, China (No. TRECKY2021-023). Written informed consent was obtained from the guardians of all the patients. The demographic and clinical characteristics of patients for bulk RNA-seq, scRNA-seq, and single-cell mass cytometry are shown in Table S2, respectively.

**Bulk RNA-seq, DEGs, and enrichment analysis**

RNA-seq was conducted by ANOROAD Corporation (Beijing, China). The DEGs and Kyoto Encyclopedia of Genes and Genomes (KEGG) pathway analysis was performed as described previously^[2]^. To explore the functions of epithelial and stromal subpopulations from the data of scRNA-seq, we performed gene set variation analysis (GSVA, v 1.40.1) using 50 hallmark pathways. All plots from bulk RNA-seq and scRNA-seq were generated using the ggplot2 (v 3.3.6) in R 4.1.0.

**Estimating the abundance of immune cell types by Immune Cell Abundance Identifier (ImmunCellAI)**

For estimating the abundance of 24 immune cell types, the log2 transformed expression data from RNA-Seq was built and uploaded to the web server for ImmunCellAI (<http://bioinfo.life.hust.edu.cn/web/ImmuCellAI/>) as previously described^[3]^.

**Single-cell suspension preparation**

Fresh tumor tissues were washed twice with PBS, cut into 1 mm^3^ piece, and enzymatically digested with the tumor dissociation kit (Miltenyi Biotec) for 1 hour on a rotor at 37°C, following the manufacturer’s instructions. The digested cells were subsequently passed through a 70 μm Cell-Strainer (BD, Biosciences) and centrifuged at 500 g for 5 minutes. After discarding the supernatant, the pelleted cells were lysed with red blood cell lysis buffer (Invitrogen) for 2 minutes on ice. Then cells were washed with PBS and centrifuged at 500 g for 5 minutes. These cells were resuspended in PBS and then used for scRNA-seq or mass cytometry. The isolation of PBMCs was conducted as previously described^[4]^.

**Single-cell RNA sequencing and data processing**

The scRNA-seq was conducted by ANOROAD (Beijing, China). The preprocessing of data including scRNA-seq data alignment, quality control, data integration, dimension reduction, major cell type annotation, cell subclustering analysis, DEGs testing, and trajectory analysis by the Monocle 3 R package were performed as previously described^[5]^.

**InferCNV analysis**

After cell annotation, the raw single-cell gene expression data of epithelial cells were extracted. We performed inferCNV analysis with the default parameter to identify malignant epithelial cells. The raw single-cell gene expression data of stromal cells was included as a control reference.

**SCENIC**

We extracted the raw UMI (unique molecular identifier) counts (genes that were expressed in 3% of samples and cells that expressed > 0 UMI) of the epithelial cells and followed the standard SCENIC procedure to identify potential transcriptional factor (TF) targets. In addition, the activity of each regulon in each cell was evaluated using AUCell^[6]^.

**Mass Cytometry**

***Antibody panel setup.*** All mass cytometry antibodies were provided by Polaris Biology, China. The details of the mass cytometry antibodies (CytoATLAS, Polaris Biology, China) are listed in Table S3.

***Sample staining and acquisition****.* Cells were washed with LunaStain cell staining buffer (Polaris Biology, China) and first stained with 10μL of Cisplatin reagent (Polaris Biology, China) at room temperature for 5 min. Cells were then washed with LunaStain cell staining buffer (Polaris Biology, China) and stained with 5μL of Fc block (Biolegend, USA) for 10 min and the heavy metal-labeled membrane antibody mixtures for 30 min at room temperature. Cells were then washed twice with LunaStain cell staining buffer (Polaris Biology, China) twice and stained with Ir-DNA intercalator reagent (Polaris Biology, China) for 10 min. After staining, cells were washed and adjusted to 1 million cells per milliliter in LunaAcq cell acquisition solution (Polaris Biology, China) together with 20μL of SureBits element calibration beads (Polaris Biology, China). Cell acquisition was performed at 500 events/ second on a mass cytometer (StarionX1, Polaris Biology, China).

**Multicolor immunohistochemistry**

We performed multicolor immunohistochemistry using the PANO 7-plex IHC Kit (Panovue, Beijing, China, Cat#0004100100). Primary antibodies used for panel 1: MMP13 (1:50, Abcam, ab219620), MMP10 (1:100, Abcam, ab261733), MCP1 (1:100, proteintech, 25542-1-AP) and CXCL14 (1:1000, Abcam, ab137541) or Panel 2: MMP13 (1:50, Abcam, ab219620), MMP10 (1:100, Abcam, ab261733), BCA1 (1:50, Abcam, ab246518) and CXCL6 (1:200, Thermofisher, PA5-67571). The primary antibodies in each panel were sequentially applied, followed by secondary antibody incubation and tyramide signal amplification (TSA). Each slide was scanned with Olympus VS200 (Olympus Germany), in conjunction with Olympus UPLXAPO 20x objective lens. Whole slide fluorescence images were analyzed with QuPath software.

**References**

1. Derkay, C.S., et al., *A staging system for assessing severity of disease and response to therapy in recurrent respiratory papillomatosis.* Laryngoscope, 1998. **108**(6): p. 935-7.

2. Wu, X., et al., *Transcriptomic Landscape of Gene Expression Profiles and Pathways in Juvenile-Onset Recurrent Respiratory Papillomatosis Tumor Tissues and Human Papillomavirus 6 and 11 E6- and E7-Overexpressing Head and Neck Squamous Cell Carcinoma Cell Lines.* J Virol, 2022. **96**(2): p. e0134221.

3. Miao, Y.R., et al., *ImmuCellAI: A Unique Method for Comprehensive T-Cell Subsets Abundance Prediction and its Application in Cancer Immunotherapy.* Adv Sci (Weinh), 2020. **7**(7): p. 1902880.

4. Wu, X., et al., *Reduced NK Cell Cytotoxicity by Papillomatosis-Derived TGF-β Contributing to Low-Risk HPV Persistence in JORRP Patients.* Front Immunol, 2022. **13**: p. 849493.

5. Wu, X., et al., *Single-cell sequencing of immune cells from anticitrullinated peptide antibody positive and negative rheumatoid arthritis.* Nat Commun, 2021. **12**(1): p. 4977.

6. Aibar, S., et al., *SCENIC: single-cell regulatory network inference and clustering.* Nat Methods, 2017. **14**(11): p. 1083-1086.
