# Supplementary material for: Investigating immune and non‐immune cellular profiles in recurrent respiratory papillomatosis by multi‐omics
Source: Clin Transl Med. 2024 Mar 1;14(3):e1570. doi: 10.1002/ctm2.1570 (PMC10905527; doi:10.1002/ctm2.1570)
Supplement: Supplementary file 2 — Supporting Information [file CTM2-14-e1570-s002.docx]

**Results**

**
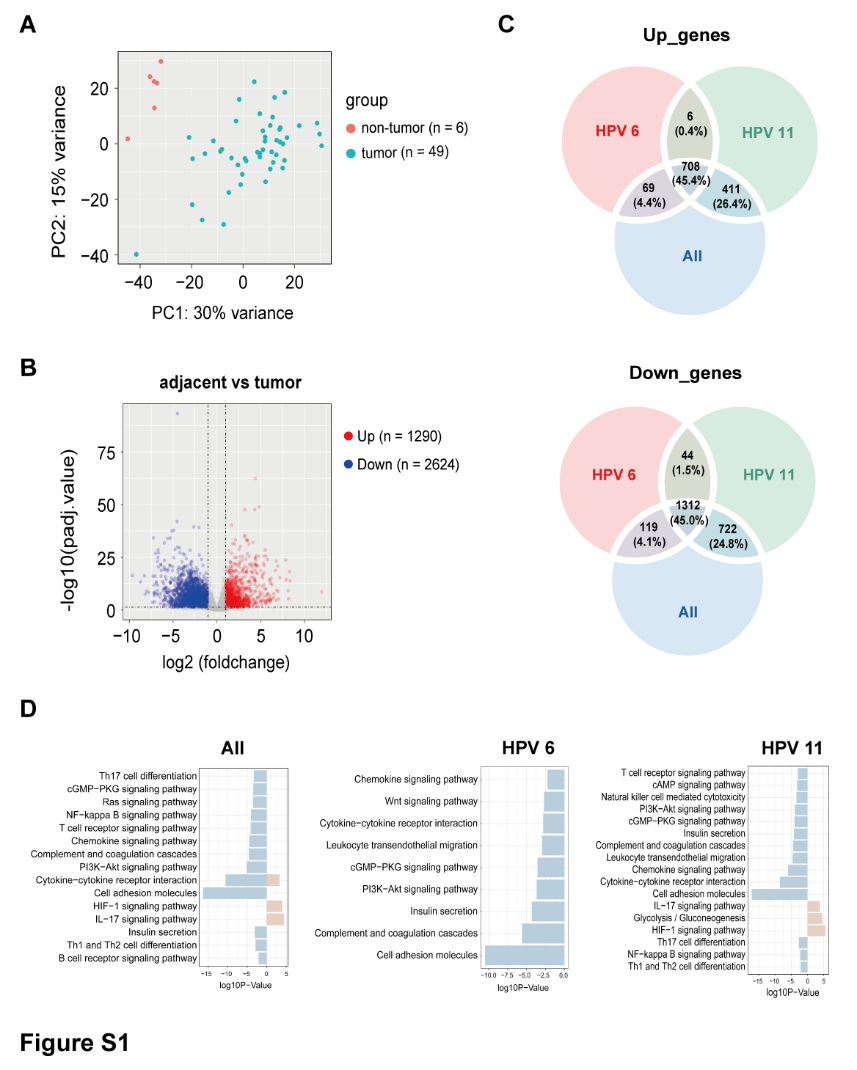
**

**Figure S1. Analysis of differentially expressed genes (DEGs) in RRP tumors.**

1. The principal component analysis (PCA) plot showed the distribution of tumor and non-tumor tissues.
2. Volcano plots of all the DEGs in tumors compared with adjacent non-tumor tissues in RRP patients. Upregulated (Up) DEGs were denoted in red, and down-regulated (Down) DEGs were denoted in blue.
3. The Venn diagram displayed the overlapped upregulated and downregulated DEGs among HPV 6, HPV 11, and all the RRP patients.
4. Kyoto Encyclopedia of Genes and Genomes (KEGG) enrichment analysis of DEGs in tumors (n=49) or different subgroups (HPV6 or HPV 11) compared with adjacent non-tumor tissues (n=6).


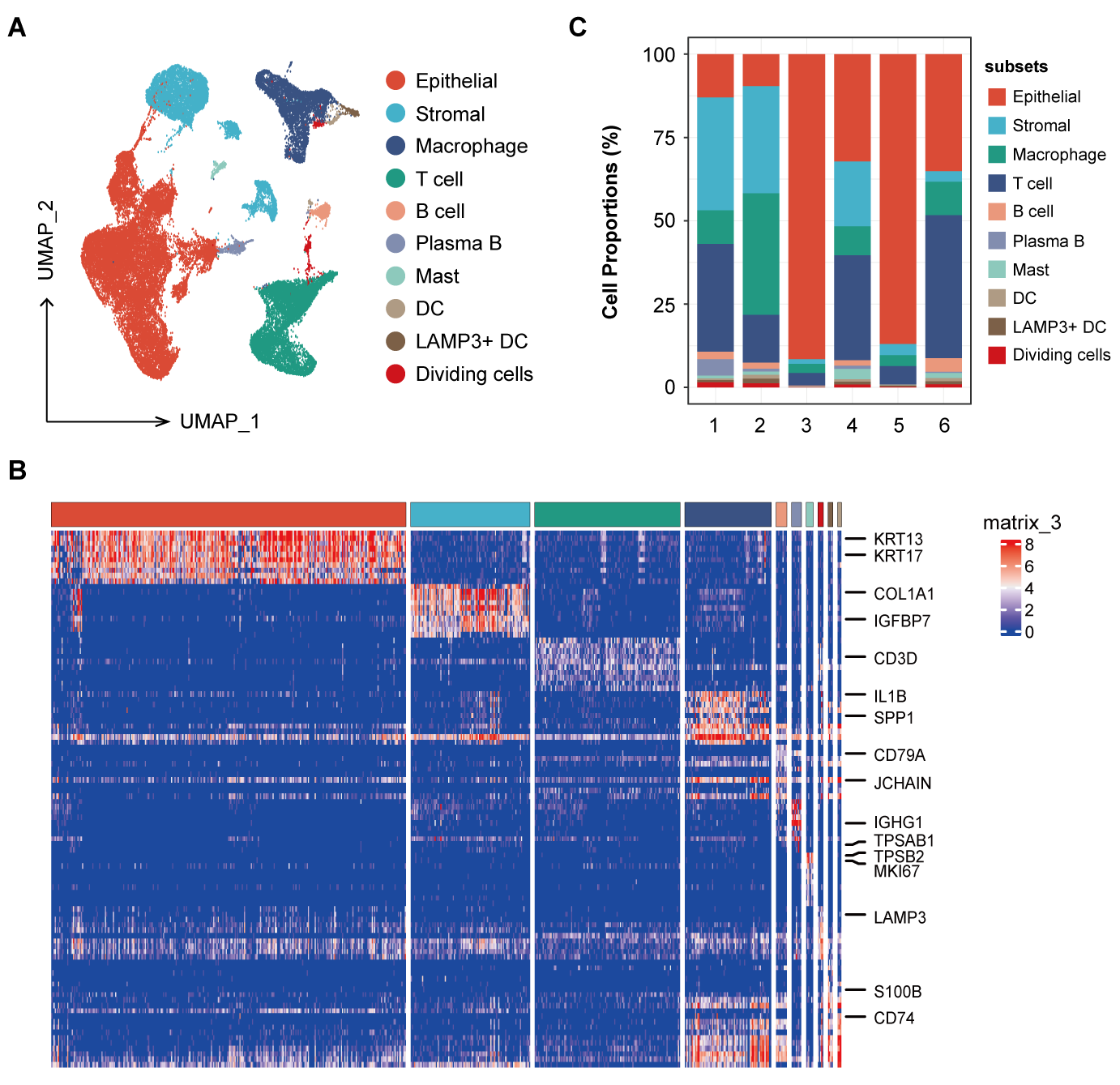


**Figure S2. Single-cell transcriptional profiling of 6 RRP tumor samples.**

1. UMAP plot shows 46,886 high-quality cells from the 10×Genomics datasets. Each dot represents a single cell colored by cell type as annotated.
2. The heatmap shows the expression of the top 10 signature genes in each cell type. Expression is indicated as the z-score normalized log_2_ level (count+1).
3. Bar plot showing the cell abundance within tumors across all samples.


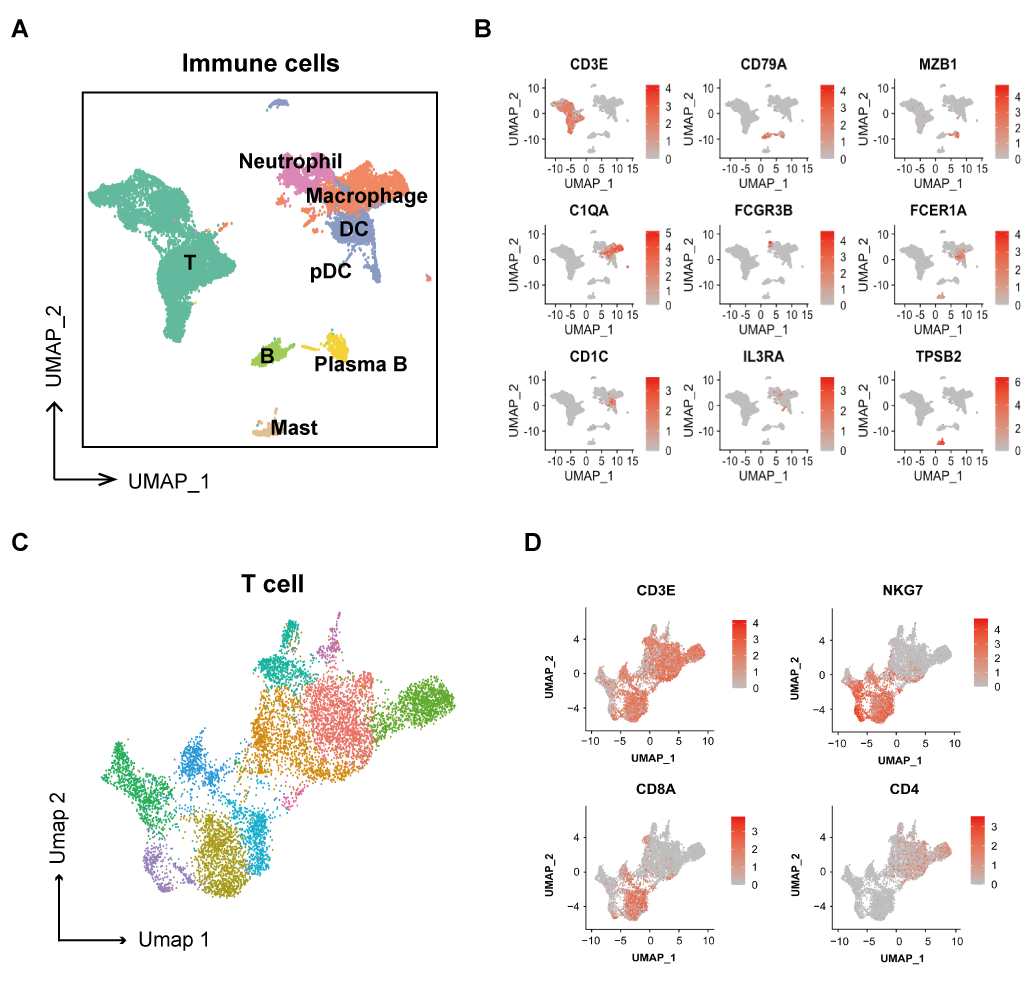


**Figure S3. Single-cell transcriptional profiling immune cells and T cell of 6 RRP tumor samples.**

1. UMAP plot shows 16,838 high-quality immune cells from the 10×Genomics datasets. Each dot represents a single cell colored by cell type as annotated.
2. Canonical cell markers were used to label immune cell types represented in the UMAP plot. The legend is labeled on a log scale.
3. UMAP plot shows 9,133 high-quality T cells from the 10×Genomics datasets. Each dot represents a single cell colored by cell type as annotated.
4. Canonical cell markers were used to label T cell types represented in the UMAP plot.


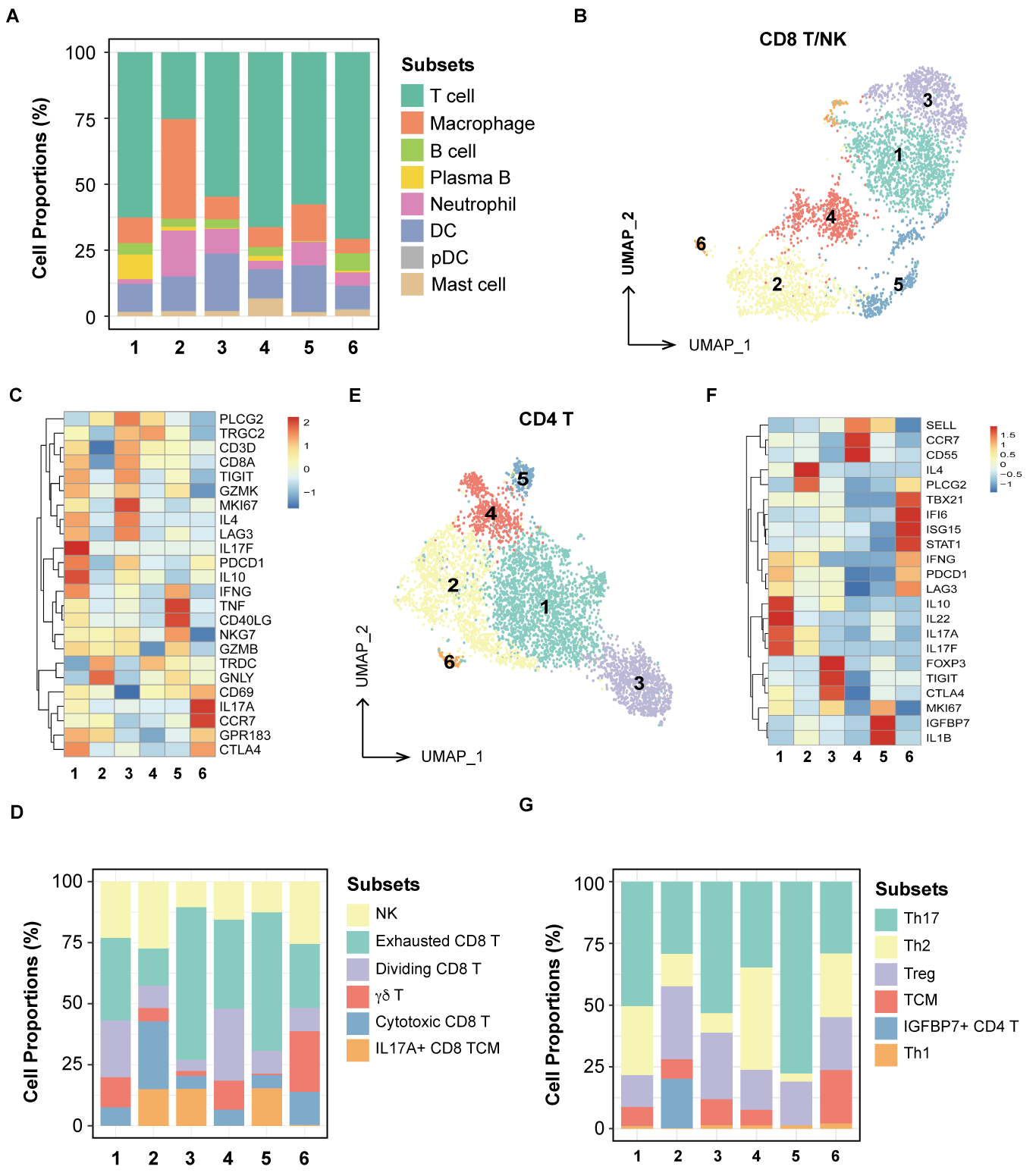


**Figure S4. Characterization of T cell populations in scRNA-seq of RRP.**

1. Bar plot showing the immune cell abundance within tumors across all samples.
2. UMAP dimensionality reduction of 3,855 CD8^+^ T/NK cells was visualized.
3. Heatmap showing interest and feature gene expression in each CD8^+^ T/NK subcluster.
4. Bar plot showing abundances of CD8^+^ T/NK subsets within tumors across all samples.
5. UMAP dimensionality reduction of 5,128 CD4^+^ T cells was visualized.
6. Heatmap showing interest and feature gene expression in each CD4^+^ T subcluster.
7. Bar plot showing abundances of CD4^+^ T subsets within tumors across all samples.


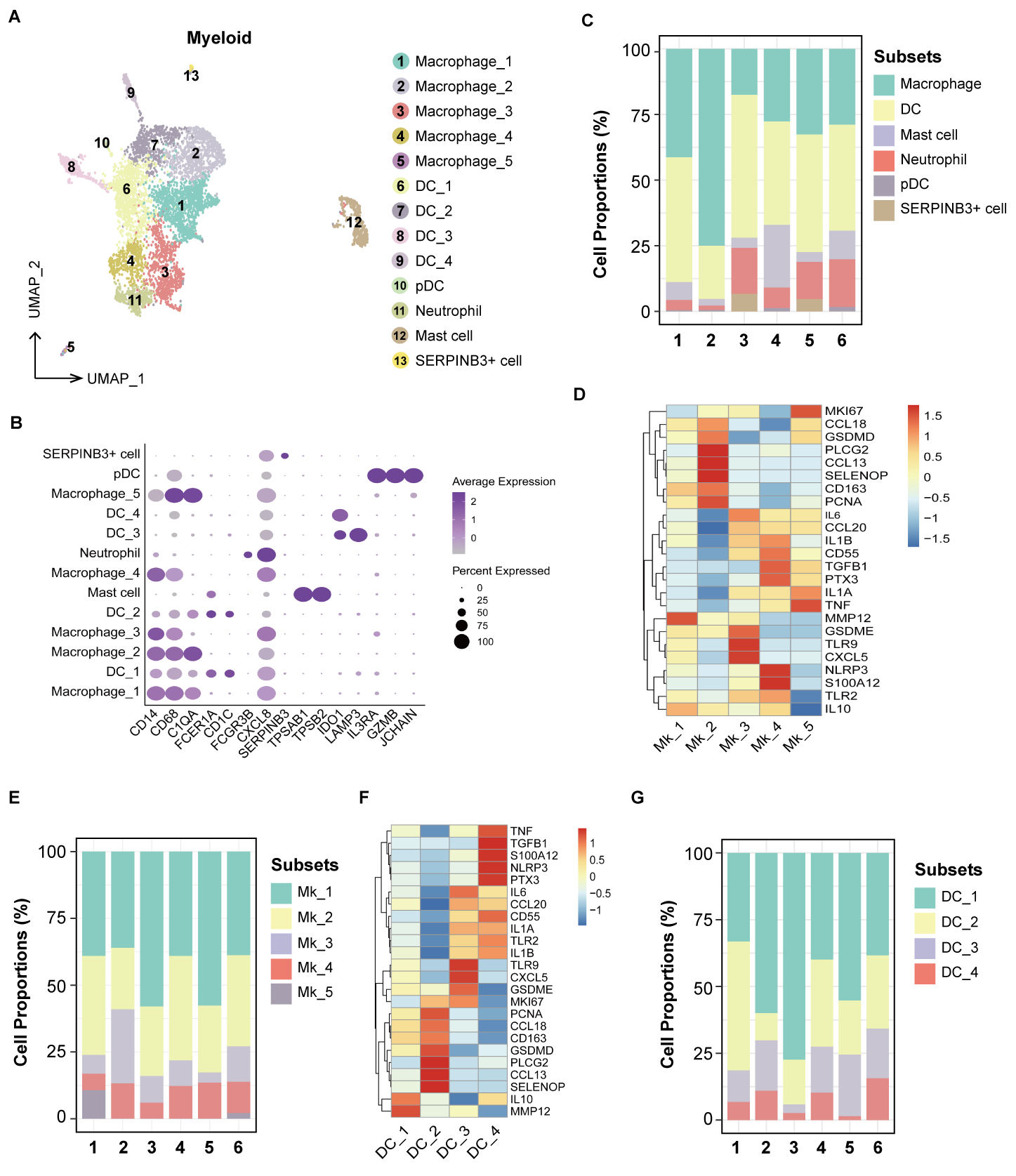


**Figure S5. Characterization of Myeloid populations in scRNA-seq of RRP.**

1. UMAP dimensionality reduction of 6,126 myeloid cells was visualized.
2. Bubble heatmap showing the selected markers annotating myeloid cell types. The size of the dot indicates the fraction of expressing cells, colored according to z-score-normalized expression levels.
3. Bar plot showing the myeloid cell abundance within tumors across all samples.
4. Heatmap showing interest and feature gene expressions in each Mk (macrophage) subcluster.
5. Bar plot showing the macrophage abundance within tumors across all samples.
6. Heatmap showing interest and feature gene expressions in each DC (dendritic cell) subcluster.
7. Bar plot showing the DC abundance within tumors across all samples.
