# Supplementary material for: Investigating immune and non‐immune cellular profiles in recurrent respiratory papillomatosis by multi‐omics
Source: Clin Transl Med. 2024 Mar 1;14(3):e1570. doi: 10.1002/ctm2.1570 (PMC10905527; doi:10.1002/ctm2.1570)
Supplement: Supplementary file 3 — Supporting Information [file CTM2-14-e1570-s001.docx]

**Tables**

**Table S1 The interested upregulated genes in RRP of the bulk RNA-seq**

| **Gene** | **Gene name** | **Log2FC** | **P.adj** |
| --- | --- | --- | --- |
| CASP14 | 14th Critical Assessment of Protein Structure Prediction | 11.96 | 2.57e-09 |
| MMP13 | Matrix Metalloproteinase 13 | 8.38 | 1.64e-14 |
| MMP10 | Matrix Metalloproteinase 10 | 8.29 | 6.59e-19 |
| KRT1 | Keratin 1 | 8.15 | 7.11e-10 |
| CA9 | Carbonic Anhydrase 9 | 7.93 | 4.60e-15 |
| CXCL8 | Chemokine (C-X-C motif) Ligand 8 | 6.12 | 1.61e-14 |
| IL36G | Interleukin 36G | 5.25 | 6.70e-16 |
| MMP1 | Matrix Metalloproteinase 1 | 5.18 | 1.38e-08 |
| CXCL6 | Chemokine (C-X-C motif) Ligand 6 | 5.11 | 1.10e-16 |
| IL1RL1 | Interleukin 1 Receptor-like 1 | 4.93 | 3.72e-08 |
| CXCL1 | Chemokine (C-X-C motif) Ligand 1 | 4.85 | 1.36e-12 |
| HLA-G | Human Leukocyte Antigen G | 4.60 | 1.94e-06 |
| SLC15A1 | Solute Carrier Family 15 Member 1 | 4.48 | 2.16e-18 |
| WNT7A | Wingless-type Protein 7A | 4.39 | 8.55e-20 |
| HK2 | Hexokinase 2 | 4.32 | 1.16e-15 |
| IL20 | Interleukin 20 | 3.67 | 0.0017 |
| SLC6A14 | Solute Carrier Family 6 Member 14 | 3.43 | 4.02e-19 |
| PFKFB4 | 6-phosphofructo-2-kinase/ Fructose-2,6-bisphosphatase 4 | 3.23 | 1.78e-16 |
| VEGFA | Vascular Endothelial Growth Factor A | 2.89 | 2.88e-19 |
| IL17C | Interleukin 17C | 2.52 | 0.0009 |
| CXCL14 | Chemokine (C-X-C motif) Ligand 14 | 2.47 | 7.87e-09 |
| GSDME | Gasdermin E | 2.25 | 1.43e-16 |

Log2FC: log2 fold change; P.adj: adjusted *P* value.

**Table S2 Demographic and clinical characteristics of included RRP patient**

|  | **Bulk RNA-seq** | **scRNA-seq** | **Mass cytometry** |
| --- | --- | --- | --- |
| **Total Number** | 49 | 6 | 24 |
| **Gender** |  |  |  |
| Male, n (%) | 21 (42.9) | 3 (50.0) | 11 (45.8) |
| Female, n (%) | 28 (57.1) | 3 (50.0) | 13 (54.2) |
| **Age (year)** | 10.46 (11.02) | 11.50 (11.55) | 12.66 (14.60) |
| **Age of onset** | 2.67 (3.25) | 3.00 (2.14) | 6.5 (10.51) |
| **Operation times** | 16 (25) | 26 (28) | 16 (19) |
| **Average interval time (months)** | 16 (6) | 146 (169) | 16 (45) |
| **Derkay score** | 15 (7) | 20 (5) | 21 (8) |
| **HPV genotyping** |  |  |  |
| HPV 6, n (%) | 6 (12.2) | 1 (16.7) | 3 (12.5) |
| HPV 11, n (%) | 13 (26.5) | - | 6 (25.0) |
| HPV 6 and HPV 11, n (%) | 30 (61.2) | 5 (83.3) | 15 (62.5) |

**Table S3 CytoATLAS mass cytometry antibodies from Polaris Bioloty**

| Category No. | Antigen | Clone | Metal |
| --- | --- | --- | --- |
| H03612094 | Anti-human CD127 | A019D5 | 141Pr |
| H32011098 | Anti-human IgD | IA6-2 | 145Nd |
| H14923099 | Anti-human CD27 | O323 | 146Nd |
| H21612105 | Anti-human CD45RO | UCHL1 | 147Sm |
| H19511107 | Anti-human CD28 | CD28.2 | 149Sm |
| H31111111 | Anti-human HLA-DR | L243 | 151Eu |
| H25754109 | Anti-human CD8 | UCHT4 | 152Sm |
| H21411112 | Anti-human CD45RA | HI100 | 153Eu |
| H03311110 | Anti-human CD123/IL-3R | 6H6 | 154Sm |
| H03112118 | Anti-human CD11c | Bu15 | 158Gd |
| H06733120 | Anti-human CD16 | 3G8 | 159Tb |
| H09212119 | Anti-human CD19 | HIB19 | 160Gd |
| H09131125 | Anti-human CD185/CXCR5 | J252D4 | 162Dy |
| H13553126 | Anti-human CD25/IL-2Rα | M-A251 | 163Dy |
| H04842127 | Anti-human CD14 | UCHM1 | 164Dy |
| H24832128 | Anti-human CD66b | 6/40C | 165Ho |
| H23414131 | Anti-human CD56 | NCAM16.2 | 166Er |
| H09813132 | Anti-human CD197/CCR7 | G043H7 | 167Er |
| H20631133 | Anti-human CD4 | OKT4 | 168Er |
| H20443134 | Anti-human CD38 | OKT10 | 170Er |
| H21231138 | Anti-human CD45 | BC8 | 171Yb |
| H40762141 | Anti-human TCRγδ | REA591 | 174Yb |
| H16712189 | Anti-human CD3 | UCHT1 | 209Bi |
| Customized | Anti-human IDO1 | 700838 | 172Yb |
| Customized | Anti-human CD15 | W6D3 | 176Yb |
| Customized | Anti-human Ki-67 | Ki-67 | 155Gd |
| Customized | Anti-human CD163 | GHI/61 | 175Lu |
| Customized | Anti-human CD117 | 104D2 | 144Nd |
| Customized | Anti-human FCƹR10 | 1F2A9 | 148Nd |
| Customized | Anti-human PLCG2 | 3A8B6 | 156Gd |
| Customized | Anti-human CD206 | 15-2 | 169Tm |
| Customized | Anti-human CD40 | 5C3 | 161Dy |
| Customized | Anti-human CTLA-4 | L3D10 | 142Nd |
| Customized | Anti-human PD-1 | EH12.2H7 | 143Nd |
| Customized | Anti-human IFN-γ | B27 | 150Nd |
| Customized | Anti-human Granzyme B | REA226 | 173Yb |
